# Supplementary material for: Dynamic spatiotemporal features in action recognition: a multimodal study
Source: Commun Biol. 2026 Apr 3;9:734. doi: 10.1038/s42003-026-09917-z (PMC13219507; doi:10.1038/s42003-026-09917-z)
Supplement: Supplementary file 1 — supplementary materials [file 42003_2026_9917_MOESM1_ESM.pdf]

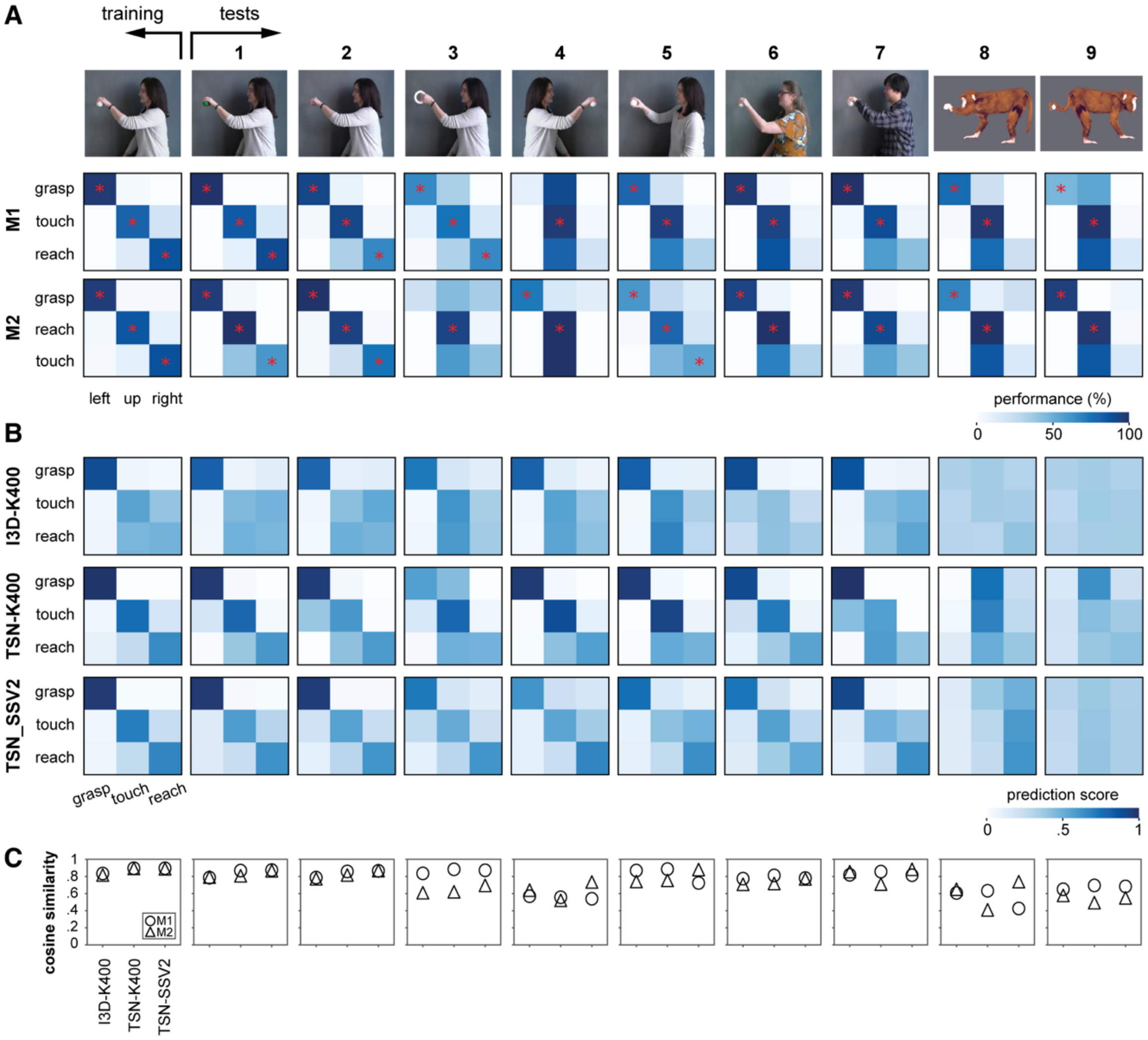

Supplementary Figure 1. **A**. Monkeys' behavioral performance and **B**. prediction scores of the CNNs for the training (1st column) and generalization tests of the categorization task. **C**. Cosine similarity between monkeys' behavioral performance and prediction scores of the CNNs for each generalization test.

Video 1. Examples of the dynamic spatial-temporal features of the action videos. Each row indicates examples of grasping (upper), touching (middle), and reaching (bottom) actions, respectively. The first column displays examples of original action videos without the object and the background. The rest of columns indicate the dynamic spatial and temporal features (of the agent) correspond to the example action videos in the first column.

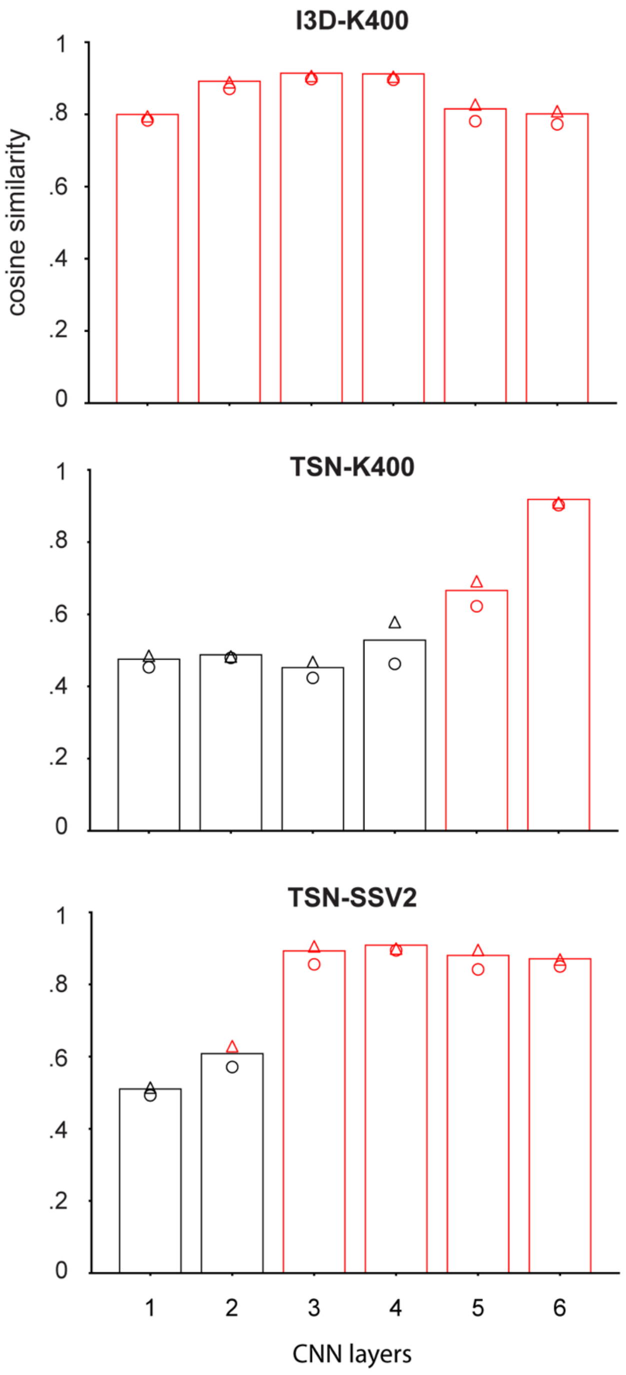

Supplementary Figure 2. Correlation between CNN layer features and monkeys' behavioral performance. Circles (monkey M1) and triangles (monkey M2) indicate individual monkeys; bar indicate group data. Red color indicates significant correlation (p<0.05, permutation approach). See Supplementary Table 5 for detailed results. Correlation between CNN layer features and monkeys' behavioral performance. Circles = monkey M1; triangles = monkey M2; bars = group data. Red marks indicate significant correlations (p < 0.05, permutation test). See Supplementary Table S5 for detailed results.

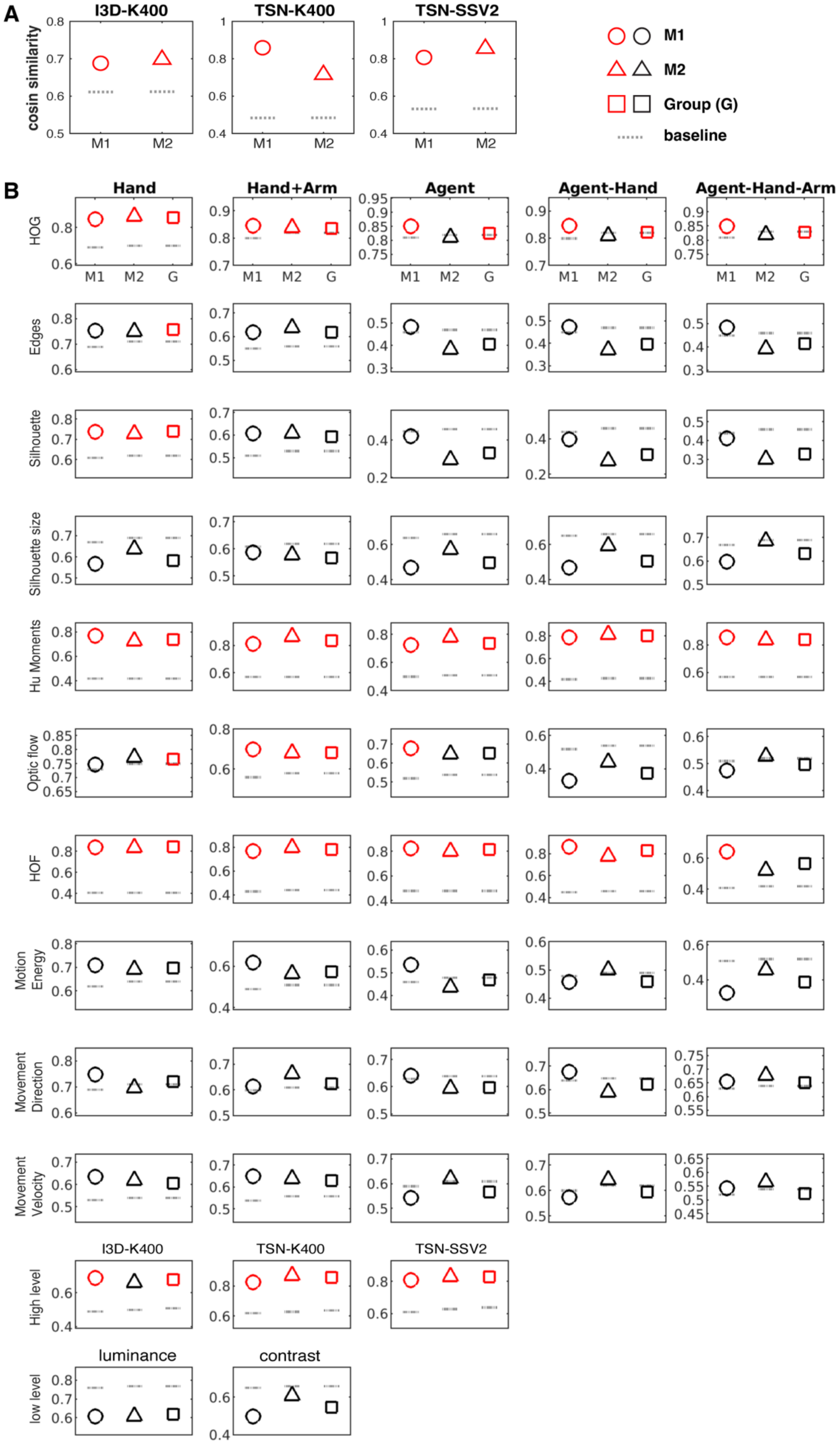
A
I3D-K400
TSN-K400
TSN-SSV2
cosine similarity
M1
M2
Group (G)
baseline
B
Hand
Hand+Arm
Agent
Agent-Hand
Agent-Hand-Arm
HOG
Edges
Silhouette
Silhouette size
Hu Moments
Optic flow
HOF
Motion Energy
Movement Direction
Movement Velocity
High level
low level
luminance
contrast

Supplementary Figure 3. Visualization of Supplementary Tables 2 (A) and 4 (B). Circles = monkey M1; triangles = monkey M2; squares = group data. Red color indicates significant correlation ($p<0.05$, permutation approach). Dashed lines indicate the corresponding baseline (mean) of the permutated tests. See Supplementary Tables 2 and 4 for detailed results.

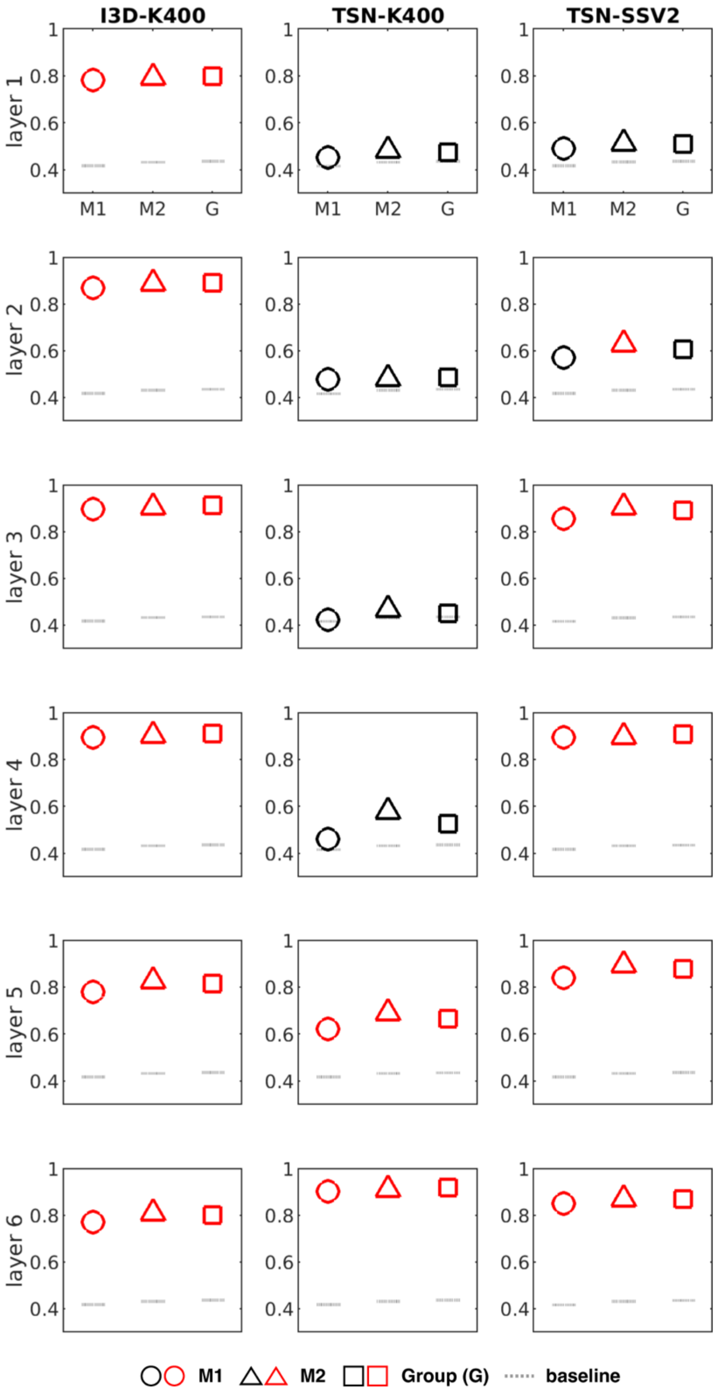

Supplementary Figure 4. Visualization of Supplementary Tables 5. Circles = monkey M1; triangles = monkey M2; squares = group data. Red color indicates significant correlation ($p<0.05$, permutation approach). Dashed lines indicate the corresponding baseline (mean) of the permutated tests. See Supplementary Tables 5 for detailed results.

Supplementary Table 1. MVPA decoding results.

ROIs		action pairs	group average	M1			M2			M3		
			accuracy	accuracy	p	p_fdr	accuracy	p	p_fdr	accuracy	p	p_fdr
V1	LH	G vs T	95,13	97,06	0,001	0,003	97,14	0,001	0,002	91,18	0,001	0,005
		G vs R	98,05	100,00	0,001	0,002	98,57	0,001	0,001	95,59	0,001	0,003
		T vs R	89,00	85,29	0,001	0,005	97,14	0,001	0,004	84,56	0,001	0,005
	RH	G vs T	79,31	81,62	0,001	0,004	85,00	0,001	0,002	71,32	0,001	0,004
		G vs R	85,94	85,29	0,001	0,002	84,29	0,001	0,002	88,24	0,001	0,003
		T vs R	66,99	52,21	0,354	0,698	67,14	0,001	0,004	81,62	0,001	0,004
V2	LH	G vs T	91,44	90,44	0,001	0,003	97,86	0,001	0,002	86,03	0,001	0,005
		G vs R	96,60	99,26	0,001	0,002	97,14	0,001	0,001	93,38	0,001	0,003
		T vs R	90,27	90,44	0,001	0,005	92,86	0,001	0,004	87,50	0,001	0,005
	RH	G vs T	79,26	72,06	0,001	0,004	90,71	0,001	0,002	75,00	0,001	0,004
		G vs R	85,40	80,15	0,001	0,002	89,29	0,001	0,002	86,76	0,001	0,003
		T vs R	71,76	58,82	0,085	0,605	80,71	0,001	0,004	75,74	0,001	0,004
V3	LH	G vs T	90,45	87,50	0,001	0,003	99,29	0,001	0,002	84,56	0,001	0,005
		G vs R	96,08	95,59	0,001	0,002	100,00	0,001	0,001	92,65	0,001	0,003
		T vs R	78,29	63,24	0,004	0,014	89,29	0,001	0,004	82,35	0,001	0,005
	RH	G vs T	81,79	89,71	0,001	0,004	82,86	0,001	0,002	72,79	0,001	0,004
		G vs R	84,20	81,62	0,001	0,002	86,43	0,001	0,002	84,56	0,001	0,003
		T vs R	63,33	54,41	0,202	0,605	65,00	0,003	0,011	70,59	0,001	0,004
V4	LH	G vs T	89,75	91,91	0,001	0,003	95,00	0,001	0,002	82,35	0,001	0,005
		G vs R	95,36	100,00	0,001	0,002	97,86	0,001	0,001	88,24	0,001	0,003
		T vs R	84,17	89,71	0,001	0,005	90,00	0,001	0,004	72,79	0,001	0,005
	RH	G vs T	81,99	82,35	0,001	0,004	87,14	0,001	0,002	76,47	0,001	0,004
		G vs R	90,10	96,32	0,001	0,002	85,00	0,001	0,002	88,97	0,001	0,003
		T vs R	78,89	88,24	0,001	0,018	77,86	0,001	0,004	70,59	0,001	0,004
MT	LH	G vs T	71,95	66,18	0,005	0,013	85,71	0,001	0,002	63,97	0,011	0,031
		G vs R	80,76	80,88	0,001	0,002	87,86	0,001	0,001	73,53	0,001	0,003
		T vs R	61,39	72,06	0,002	0,008	62,86	0,017	0,059	49,26	0,590	0,620
	RH	G vs T	70,48	64,71	0,005	0,011	85,71	0,001	0,002	61,03	0,034	0,054
		G vs R	66,20	60,29	0,036	0,050	72,14	0,001	0,002	66,18	0,002	0,004
		T vs R	58,39	52,94	0,337	0,698	69,29	0,001	0,004	52,94	0,319	0,521
MST	LH	G vs T	63,36	63,97	0,012	0,028	62,14	0,021	0,029	63,97	0,011	0,031
		G vs R	69,65	77,94	0,001	0,002	70,71	0,002	0,003	60,29	0,048	0,059
		T vs R	59,80	71,32	0,001	0,005	50,71	0,449	0,471	57,35	0,093	0,163
FST	LH	G vs T	67,30	62,50	0,020	0,038	85,71	0,001	0,002	53,68	0,299	0,369
		G vs R	75,13	77,94	0,001	0,002	86,43	0,001	0,001	61,03	0,042	0,055
		T vs R	58,05	61,76	0,031	0,081	54,29	0,242	0,316	58,09	0,092	0,163
	RH	G vs T	69,28	66,91	0,004	0,010	83,57	0,001	0,002	57,35	0,110	0,116
		G vs R	70,52	69,85	0,001	0,002	82,14	0,001	0,002	59,56	0,059	0,066
		T vs R	54,32	55,15	0,183	0,605	59,29	0,059	0,177	48,53	0,648	0,729
STPm	LH	G vs T	58,38	56,62	0,128	0,188	70,71	0,001	0,002	47,79	0,702	0,702
		G vs R	63,68	57,35	0,121	0,141	79,29	0,001	0,001	54,41	0,226	0,250
		T vs R	53,39	54,41	0,229	0,300	54,29	0,254	0,316	51,47	0,430	0,596
	RH	G vs T	62,30	55,88	0,134	0,201	70,71	0,001	0,002	60,29	0,031	0,054
		G vs R	64,06	68,38	0,001	0,002	65,71	0,006	0,008	58,09	0,083	0,088
		T vs R	48,76	47,79	0,686	0,835	51,43	0,442	0,673	47,06	0,717	0,759
STPa	LH	G vs T	57,74	56,62	0,143	0,188	60,71	0,040	0,049	55,88	0,162	0,227
		G vs R	65,71	61,03	0,040	0,052	72,14	0,001	0,001	63,97	0,009	0,019
		T vs R	54,43	56,62	0,106	0,171	47,86	0,631	0,631	58,82	0,083	0,163
	RH	G vs T	55,99	52,94	0,303	0,341	63,57	0,014	0,017	51,47	0,439	0,439
		G vs R	53,65	53,68	0,301	0,318	52,86	0,340	0,340	54,41	0,267	0,267
		T vs R	49,02	46,32	0,731	0,835	50,00	0,512	0,673	50,74	0,490	0,610
ML	LH	G vs T	75,42	84,56	0,001	0,003	82,14	0,001	0,002	59,56	0,038	0,072
		G vs R	83,99	88,97	0,001	0,002	82,86	0,001	0,001	80,15	0,001	0,003
		T vs R	64,71	58,09	0,103	0,171	74,29	0,001	0,004	61,76	0,012	0,045
	RH	G vs T	73,91	75,74	0,001	0,004	86,43	0,001	0,002	59,56	0,039	0,054
		G vs R	76,63	80,15	0,001	0,002	83,57	0,001	0,002	66,18	0,002	0,004
		T vs R	55,82	49,26	0,607	0,835	56,43	0,138	0,310	61,76	0,016	0,058
MSB	LH	G vs T	73,47	77,94	0,001	0,003	81,43	0,001	0,002	61,03	0,009	0,031
		G vs R	74,64	71,32	0,001	0,002	86,43	0,001	0,001	66,18	0,001	0,003
		T vs R	53,86	55,88	0,139	0,208	56,43	0,087	0,183	49,26	0,576	0,620
	RH	G vs T	66,94	66,91	0,002	0,006	72,14	0,001	0,002	61,76	0,010	0,026
		G vs R	70,06	69,85	0,001	0,002	79,29	0,001	0,002	61,03	0,014	0,019
		T vs R	52,91	55,15	0,181	0,605	53,57	0,290	0,579	50,00	0,508	0,610
AL	LH	G vs T	62,04	63,24	0,014	0,029	71,43	0,001	0,002	51,47	0,422	0,467
		G vs R	61,10	55,15	0,208	0,230	67,86	0,001	0,001	60,29	0,034	0,048
		T vs R	52,44	47,79	0,645	0,702	51,43	0,422	0,466	58,09	0,055	0,128
	RH	G vs T	72,23	64,71	0,007	0,014	82,14	0,001	0,002	69,85	0,001	0,004
		G vs R	69,85	68,38	0,003	0,006	75,00	0,001	0,002	66,18	0,002	0,004
		T vs R	49,54	44,12	0,857	0,908	47,14	0,722	0,765	57,35	0,100	0,225
ASB	LH	G vs T	59,14	51,47	0,407	0,427	67,86	0,002	0,004	58,09	0,097	0,157
		G vs R	66,20	62,50	0,017	0,024	72,86	0,001	0,001	63,24	0,011	0,021
		T vs R	59,22	58,09	0,088	0,168	59,29	0,055	0,128	60,29	0,035	0,092
	RH	G vs T	61,08	52,21	0,391	0,414	70,00	0,001	0,002	61,03	0,038	0,054
		G vs R	65,41	57,35	0,107	0,120	77,86	0,001	0,002	61,03	0,046	0,055
		T vs R	43,86	39,71	0,957	0,957	51,43	0,459	0,673	40,44	0,955	0,955
TEr	LH	G vs T	53,17	55,88	0,139	0,188	52,14	0,382	0,401	51,47	0,423	0,467
		G vs R	60,11	58,09	0,079	0,097	69,29	0,001	0,001	52,94	0,294	0,308
		T vs R	52,90	53,68	0,260	0,321	54,29	0,230	0,316	50,74	0,468	0,596
	RH	G vs T	59,20	55,15	0,211	0,292	61,43	0,022	0,025	61,03	0,023	0,046
		G vs R	63,37	61,03	0,036	0,050	61,43	0,031	0,033	67,65	0,002	0,004
		T vs R	51,23	52,21	0,388	0,698	50,00	0,523	0,673	51,47	0,436	0,603
AIP	LH	G vs T	62,35	66,18	0,002	0,006	65,71	0,005	0,009	55,15	0,156	0,227
		G vs R	68,19	72,06	0,001	0,002	69,29	0,002	0,003	63,24	0,015	0,026
		T vs R	59,19	50,74	0,487	0,568	62,86	0,026	0,078	63,97	0,013	0,045
	RH	G vs T	57,23	51,47	0,458	0,458	62,14	0,014	0,017	58,09	0,071	0,080
		G vs R	64,33	60,29	0,044	0,057	63,57	0,005	0,007	69,12	0,001	0,003
		T vs R	50,02	48,53	0,610	0,835	47,86	0,681	0,765	53,68	0,286	0,514
PFG	LH	G vs T	59,45	55,15	0,198	0,226	61,43	0,026	0,034	61,76	0,026	0,057
		G vs R	64,99	66,91	0,002	0,003	70,71	0,001	0,001	57,35	0,104	0,121
		T vs R	53,65	58,82	0,070	0,147	52,86	0,328	0,382	49,26	0,564	0,620
S2	LH	G vs T	54,88	55,88	0,154	0,190	52,14	0,367	0,401	56,62	0,086	0,150
		G vs R	56,36	53,68	0,279	0,293	51,43	0,402	0,402	63,97	0,005	0,012
		T vs R	59,51	62,50	0,017	0,051	55,00	0,175	0,306	61,03	0,015	0,045
	RH	G vs T	57,78	54,41	0,252	0,324	56,43	0,144	0,144	62,50	0,017	0,038
		G vs R	60,36	47,06	0,721	0,721	67,86	0,001	0,002	66,18	0,005	0,008
		T vs R	56,79	55,88	0,173	0,605	57,14	0,094	0,241	57,35	0,095	0,225
S1	LH	G vs T	52,44	55,15	0,205	0,226	51,43	0,453	0,453	50,74	0,491	0,515
		G vs R	50,70	46,32	0,718	0,718	53,57	0,288	0,302	52,21	0,401	0,401
		T vs R	51,41	47,06	0,668	0,702	56,43	0,141	0,269	50,74	0,483	0,596
F5	LH	G vs T	60,18	59,56	0,046	0,074	61,43	0,015	0,024	59,56	0,027	0,057
		G vs R	63,87	63,24	0,006	0,010	60,71	0,017	0,021	67,65	0,001	0,003
		T vs R	51,68	47,06	0,724	0,724	53,57	0,256	0,316	54,41	0,200	0,323
	RH	G vs T	58,98	60,29	0,024	0,043	59,29	0,039	0,041	57,35	0,065	0,078
		G vs R	63,61	60,29	0,023	0,038	62,14	0,015	0,017	68,38	0,001	0,003
		T vs R	54,63	52,21	0,387	0,698	52,86	0,329	0,592	58,82	0,051	0,153
45B	LH	G vs T	60,25	61,76	0,032	0,056	55,00	0,198	0,231	63,97	0,012	0,031
		G vs R	64,59	70,59	0,001	0,002	62,14	0,024	0,028	61,03	0,025	0,037
		T vs R	54,37	55,15	0,208	0,291	54,29	0,240	0,316	53,68	0,277	0,415
	RH	G vs T	60,41	52,94	0,301	0,341	62,86	0,008	0,011	65,44	0,004	0,012
		G vs R	64,33	64,71	0,012	0,022	62,86	0,012	0,014	65,44	0,007	0,010
		T vs R	46,88	46,32	0,742	0,835	43,57	0,897	0,897	50,74	0,426	0,603
46v	LH	G vs T	55,02	48,53	0,642	0,642	62,86	0,019	0,028	53,68	0,270	0,354
		G vs R	59,52	62,50	0,017	0,024	54,29	0,237	0,262	61,76	0,021	0,034
		T vs R	55,29	59,56	0,067	0,147	60,71	0,044	0,115	45,59	0,772	0,772
	RH	G vs T	61,61	58,82	0,084	0,137	65,71	0,003	0,004	60,29	0,047	0,060
		G vs R	63,05	58,82	0,078	0,094	69,29	0,003	0,004	61,03	0,042	0,054
		T vs R	51,24	50,74	0,469	0,767	48,57	0,624	0,749	54,41	0,216	0,432

Note: G – grasping, T – touching, R – reaching.

Supplementary Table 2. Cosine similarity between monkey and CNN performance with baselines derived from permutation tests

	M1			M2		
	**cos sim**	**p**	**base**	**cos sim**	**p**	**base**
**I3D-K400**	0,688	**0,041**	0,611	0,698	**0,026**	0,612
**TSN-K400**	0,859	**<0,001**	0,483	0,715	**<0,001**	0,484
**TSN-SSV2**	0,807	**<0,001**	0,532	0,855	**<0,001**	0,533

Supplementary Table 3. Chamfer distance of features.

	Features	Masks \ Generalization tests	Grasping vs Touching 1	2	3	4	5	6	7	8	9	Grasping vs Reaching 1	2	3	4	5	6	7	8	9	Touching vs Reaching 1	2	3	4	5	6	7	8	9
spatial	HOG	Hand	0,291	0,273	0,214	0,279	0,223	0,326	0,298	0,051	0,280	0,378	0,337	0,413	0,417	0,354	0,391	0,379	0,050	0,280	0,185	0,205	0,172	0,174	0,125	0,123	0,125	0,048	0,009
		Hand + Arm	0,178	0,188	0,181	0,182	0,197	0,208	0,197	0,054	0,126	0,203	0,224	0,255	0,265	0,258	0,258	0,207	0,051	0,124	0,133	0,139	0,143	0,132	0,125	0,168	0,134	0,024	0,030
		Agent	0,167	0,177	0,171	0,167	0,114	0,189	0,168	0,047	0,028	0,175	0,194	0,220	0,208	0,134	0,215	0,177	0,044	0,028	0,121	0,122	0,125	0,123	0,102	0,148	0,150	0,028	0,012
		Agent – Hand	0,175	0,189	0,179	0,180	0,123	0,194	0,182	0,049	0,026	0,184	0,206	0,225	0,222	0,145	0,224	0,190	0,046	0,026	0,126	0,126	0,134	0,133	0,114	0,156	0,168	0,029	0,012
		Agent – Hand – Arm	0,165	0,188	0,162	0,172	0,134	0,187	0,164	0,048	0,027	0,179	0,199	0,206	0,201	0,159	0,218	0,173	0,046	0,027	0,119	0,118	0,118	0,126	0,129	0,151	0,164	0,029	0,014
	Edges	Hand	0,232	0,291	0,259	0,342	0,212	0,417	1,065	0,125	0,192	1,001	0,705	0,871	1,077	0,605	0,831	0,968	0,279	0,365	0,252	0,240	0,268	0,164	0,134	0,197	0,040	0,078	0,058
		Hand + Arm	0,035	0,023	0,063	0,070	0,048	0,023	0,069	0,008	0,035	0,125	0,100	0,174	0,237	0,141	0,066	0,074	0,020	0,045	0,034	0,034	0,028	0,028	0,043	0,016	0,008	0,007	0,004
		Agent	0,005	0,005	0,043	0,021	0,004	0,004	0,015	0,001	0,001	0,012	0,027	0,105	0,069	0,025	0,014	0,010	0,002	0,002	0,008	0,017	0,009	0,009	0,017	0,006	0,004	0,001	0,000
		Agent – Hand	0,006	0,006	0,038	0,018	0,003	0,003	0,009	0,001	0,001	0,010	0,025	0,092	0,059	0,019	0,013	0,005	0,001	0,001	0,006	0,014	0,006	0,007	0,014	0,005	0,004	0,000	0,000
		Agent – Hand – Arm	0,006	0,008	0,033	0,019	0,003	0,003	0,012	0,001	0,000	0,010	0,024	0,085	0,057	0,014	0,013	0,006	0,001	0,000	0,006	0,012	0,005	0,008	0,012	0,005	0,004	0,000	0,000
	Silouhette	Hand	0,230	0,322	0,314	0,345	0,184	0,595	1,510	0,109	0,181	1,174	0,895	1,014	1,225	0,563	1,352	1,350	0,249	0,309	0,293	0,343	0,269	0,167	0,108	0,163	0,027	0,078	0,032
		Hand + Arm	0,018	0,011	0,053	0,044	0,031	0,017	0,061	0,007	0,031	0,072	0,066	0,144	0,163	0,110	0,056	0,065	0,016	0,045	0,018	0,023	0,017	0,015	0,031	0,010	0,005	0,005	0,003
		Agent	0,001	0,001	0,038	0,014	0,001	0,002	0,012	0,000	0,001	0,005	0,019	0,097	0,053	0,016	0,012	0,007	0,001	0,002	0,005	0,011	0,005	0,004	0,010	0,004	0,002	0,000	0,000
		Agent – Hand	0,001	0,001	0,031	0,012	0,000	0,001	0,007	0,000	0,000	0,004	0,016	0,081	0,047	0,012	0,010	0,003	0,000	0,000	0,003	0,009	0,003	0,003	0,008	0,003	0,002	0,000	0,000
		Agent – Hand – Arm	0,003	0,002	0,029	0,012	0,000	0,001	0,008	0,000	0,000	0,005	0,018	0,076	0,047	0,009	0,010	0,003	0,000	0,000	0,003	0,009	0,003	0,004	0,007	0,003	0,002	0,000	0,000
	Silouhette size	Hand	0,029	0,259	0,289	0,192	0,106	0,430	0,285	0,204	0,552	0,081	0,171	0,455	0,459	0,116	0,217	0,184	0,227	0,914	0,123	0,065	0,031	0,080	0,084	0,379	0,055	0,020	0,047
		Hand + Arm	0,033	0,054	0,250	0,072	0,044	0,032	0,237	0,355	0,206	0,085	0,178	0,285	0,206	0,178	0,089	0,162	0,222	0,502	0,054	0,067	0,079	0,039	0,082	0,028	0,048	0,033	0,029
		Agent	0,062	0,051	0,266	0,491	0,092	0,098	0,216	0,076	0,764	0,142	0,182	0,677	0,733	0,312	0,377	0,189	0,076	0,886	0,048	0,051	0,073	0,035	0,102	0,085	0,015	0,007	0,090
		Agent – Hand	0,095	0,097	0,244	0,640	0,124	0,083	0,126	0,065	0,593	0,242	0,291	0,664	1,054	0,389	0,376	0,122	0,066	0,849	0,074	0,070	0,100	0,062	0,122	0,110	0,015	0,004	0,013
		Agent – Hand – Arm	0,358	0,282	0,192	1,055	0,320	0,450	0,186	0,044	0,120	0,981	0,795	0,753	1,761	0,738	0,965	0,117	0,051	0,389	0,223	0,169	0,329	0,104	0,188	0,344	0,073	0,003	0,137
	Hu Moments	Hand	0,365	1,313	2,084	1,397	0,812	1,380	1,434	0,280	0,693	0,805	1,557	2,419	1,081	0,311	1,293	1,303	0,216	1,284	0,680	0,630	0,037	0,220	0,594	0,657	0,334	0,022	0,201
		Hand + Arm	0,599	0,526	0,187	0,572	0,899	0,465	1,040	0,489	0,489	1,241	0,405	0,376	0,558	0,677	0,731	0,448	0,371	0,416	0,509	0,261	0,067	0,049	0,462	0,536	0,538	0,764	0,575
		Agent	0,512	0,467	0,544	0,823	0,860	1,356	0,709	1,129	1,439	0,727	0,672	0,850	0,945	0,551	1,370	0,664	0,656	1,462	0,199	0,163	0,612	0,394	0,390	0,177	0,053	0,798	0,352
		Agent – Hand	1,419	0,867	1,144	1,433	1,206	1,545	0,773	0,355	0,914	1,688	1,691	1,586	1,904	1,310	1,734	0,901	0,421	0,810	0,154	0,429	0,283	0,190	0,264	0,150	0,031	0,115	0,189
		Agent – Hand – Arm	0,779	0,573	0,695	0,451	0,784	0,357	0,453	0,249	0,960	0,784	1,025	0,879	0,711	1,173	0,787	0,486	0,622	0,945	0,897	0,900	0,825	0,382	0,285	0,712	0,344	0,820	0,132
temporal	Optic Flow	Hand	0,145	0,120	0,108	0,174	0,099	0,261	0,277	0,033	0,079	0,166	0,216	0,225	0,260	0,133	0,164	0,351	0,065	0,168	0,033	0,072	0,026	0,025	0,048	0,036	0,032	0,023	0,018
		Hand + Arm	0,029	0,021	0,046	0,045	0,052	0,026	0,085	0,010	0,030	0,045	0,060	0,115	0,086	0,071	0,041	0,082	0,016	0,062	0,009	0,019	0,014	0,010	0,023	0,011	0,009	0,005	0,006
		Agent	0,022	0,017	0,036	0,036	0,041	0,017	0,041	0,005	0,019	0,034	0,050	0,100	0,067	0,046	0,026	0,044	0,010	0,048	0,006	0,013	0,009	0,006	0,008	0,008	0,007	0,002	0,004
		Agent – Hand	0,020	0,009	0,031	0,016	0,026	0,009	0,035	0,008	0,447	0,040	0,041	0,100	0,051	0,075	0,028	0,029	0,009	0,162	0,006	0,012	0,011	0,006	0,010	0,007	0,005	0,001	0,006
		Agent – Hand – Arm	0,087	0,053	0,078	0,032	0,864	0,064	0,020	0,037	0,546	0,149	0,128	0,186	0,071	0,820	0,237	0,018	0,050	0,546	0,004	0,021	0,014	0,007	0,005	0,030	0,008	0,022	0,000
	HOF	Hand	1,525	1,166	1,302	0,880	0,851	1,242	1,939	0,513	1,383	1,665	1,356	0,922	0,702	0,868	1,361	1,944	0,570	1,408	0,024	0,150	0,455	0,066	0,014	0,306	0,007	0,168	0,082
		Hand + Arm	0,845	0,607	0,688	0,572	0,833	0,839	1,086	0,839	1,446	0,844	0,590	0,690	0,589	0,857	0,723	1,000	1,040	1,459	0,017	0,032	0,240	0,022	0,011	0,020	0,011	0,091	0,050
		Agent	0,840	0,582	0,691	0,725	0,646	0,828	0,911	0,828	0,373	0,838	0,577	0,675	0,578	0,639	0,673	0,872	1,017	0,461	0,015	0,020	0,227	0,176	0,008	0,015	0,012	0,094	0,030
		Agent – Hand	0,841	0,581	0,693	0,188	0,641	0,830	0,901	0,829	0,355	0,837	0,578	0,692	0,036	0,640	0,658	0,868	1,012	0,421	0,018	0,022	0,214	0,185	0,009	0,017	0,013	0,103	0,035
		Agent – Hand – Arm	0,054	0,041	0,901	0,194	0,605	0,775	0,844	0,319	0,320	0,074	0,047	0,902	0,039	0,594	0,342	0,832	0,352	0,402	0,030	0,026	0,028	0,192	0,013	0,036	0,018	0,220	0,049
	Motion Energy	Hand	0,035	0,052	0,088	0,071	0,021	0,065	0,222	0,021	0,057	0,091	0,135	0,214	0,167	0,094	0,093	0,213	0,040	0,107	0,033	0,062	0,021	0,023	0,046	0,022	0,009	0,006	0,007
		Hand + Arm	0,009	0,007	0,033	0,024	0,008	0,011	0,053	0,004	0,011	0,030	0,035	0,105	0,076	0,040	0,040	0,042	0,004	0,017	0,010	0,015	0,011	0,011	0,022	0,009	0,005	0,001	0,001
		Agent	0,007	0,003	0,028	0,017	0,002	0,005	0,019	0,001	0,003	0,024	0,027	0,092	0,057	0,014	0,024	0,013	0,001	0,005	0,006	0,010	0,008	0,007	0,007	0,005	0,003	0,000	0,000
		Agent – Hand	0,006	0,003	0,026	0,016	0,004	0,005	0,017	0,003	0,108	0,021	0,025	0,088	0,055	0,012	0,024	0,011	0,001	0,061	0,006	0,009	0,008	0,007	0,007	0,005	0,003	0,000	0,000
		Agent – Hand – Arm	0,083	0,011	0,023	0,010	0,072	0,022	0,006	0,000	0,615	0,059	0,070	0,089	0,038	0,200	0,077	0,004	0,000	0,615	0,003	0,009	0,005	0,004	0,007	0,004	0,002	0,000	0,000
	Movement Direction	Hand	0,307	0,126	0,261	0,338	0,803	0,240	0,321	0,072	0,035	0,548	0,248	0,528	0,411	0,402	0,303	0,243	0,068	0,041	0,239	0,077	0,212	0,080	0,198	0,083	0,045	0,035	0,020
		Hand + Arm	0,024	0,103	0,143	0,355	0,054	0,179	0,270	0,025	0,029	0,116	0,261	0,311	0,344	0,132	0,197	0,251	0,021	0,038	0,090	0,071	0,028	0,124	0,114	0,041	0,066	0,014	0,014
		Agent	0,104	0,116	0,207	0,198	0,019	0,111	0,355	0,042	0,063	0,056	0,257	0,554	0,425	0,172	0,185	0,267	0,058	0,073	0,155	0,078	0,111	0,136	0,177	0,141	0,027	0,056	0,035
		Agent – Hand	0,058	0,149	0,337	0,176	0,085	0,288	0,198	0,039	0,035	0,222	0,330	0,597	0,393	0,137	0,331	0,086	0,075	0,054	0,232	0,066	0,069	0,082	0,217	0,109	0,045	0,033	0,030
		Agent – Hand – Arm	0,056	0,216	0,203	0,281	0,068	0,296	0,306	0,026	0,035	0,013	0,168	0,356	0,396	0,191	0,317	0,162	0,018	0,036	0,085	0,262	0,041	0,172	0,135	0,061	0,123	0,011	0,026
	Movement Velocity	Hand	0,039	0,013	0,099	0,088	0,022	0,033	0,083	0,045	0,041	0,093	0,091	0,267	0,242	0,109	0,124	0,108	0,042	0,138	0,035	0,039	0,040	0,039	0,050	0,032	0,019	0,001	0,024
		Hand + Arm	0,039	0,042	0,104	0,078	0,028	0,051	0,100	0,027	0,058	0,124	0,108	0,279	0,216	0,105	0,142	0,140	0,005	0,255	0,054	0,026	0,036	0,034	0,048	0,026	0,019	0,009	0,029
		Agent	0,030	0,020	0,120	0,157	0,050	0,065	0,325	0,008	0,487	0,083	0,112	0,321	0,268	0,176	0,260	0,367	0,009	0,560	0,044	0,047	0,047	0,024	0,067	0,072	0,014	0,005	0,003
		Agent – Hand	0,019	0,031	0,074	0,210	0,078	0,012	0,342	0,021	0,039	0,117	0,163	0,242	0,370	0,211	0,237	0,392	0,021	0,539	0,076	0,060	0,074	0,030	0,048	0,177	0,013	0,004	0,097
		Agent – Hand – Arm	0,017	0,009	0,085	0,079	0,011	0,041	0,092	0,020	0,021	0,059	0,070	0,213	0,230	0,071	0,126	0,073	0,023	0,175	0,044	0,034	0,045	0,040	0,058	0,028	0,010	0,003	0,192
high-level	I3D-K400		1,409	1,273	1,243	1,287	1,442	1,366	1,342	0,111	0,118	1,425	1,412	1,170	1,177	1,428	1,229	1,464	0,137	0,144	0,344	0,458	0,310	0,310	0,290	0,442	0,300	0,047	0,038
	TSN-K400		0,834	0,699	0,413	0,187	0,892	0,529	0,857	0,103	0,072	0,926	0,994	0,428	0,164	0,877	0,290	0,827	0,097	0,099	0,076	0,241	0,036	0,010	0,014	0,266	0,045	0,053	0,073
	TSN-SSV2		1,108	0,992	0,549	0,218	1,033	0,752	0,874	0,069	0,089	1,074	1,081	0,677	0,198	1,061	0,596	0,886	0,105	0,088	0,064	0,070	0,053	0,035	0,031	0,162	0,040	0,048	0,029
low-level	luminance		0,067	1,932	0,195	0,264	0,078	0,032	0,552	0,187	0,018	0,209	2,133	0,567	0,365	0,272	0,393	0,329	0,159	0,016	0,348	0,083	0,183	0,132	0,137	0,455	1,551	0,101	0,011
	contrast		0,227	0,115	0,234	0,107	0,088	0,410	0,705	0,145	1,610	0,210	0,244	0,415	0,315	0,383	0,274	0,604	0,120	1,652	0,033	0,061	0,059	0,067	0,503	0,148	0,040	0,011	0,002

Supplementary Table 4. Cosine similarity between monkey behavior and features with baselines derived from permutation tests.

Features		Masks	M1			M2			Group		
			cos sim	p	base	cos sim	p	base	cos sim	p	base
spatial	HOG	Hand	0,850	**0,001**	0,690	0,864	**<0,001**	0,700	0,857	**<0,001**	0,700
		Hand + Arm	0,847	**0,020**	0,800	0,839	**0,039**	0,820	0,837	**0,005**	0,820
		Agent	0,852	**0,021**	0,810	0,811	0,144	0,820	0,826	**0,019**	0,820
		Agent – Hand	0,848	**0,017**	0,800	0,810	0,115	0,820	0,823	**0,018**	0,820
		Agent – Hand – Arm	0,850	**0,022**	0,810	0,821	0,109	0,830	0,830	**0,019**	0,830
	Edges	Hand	0,755	0,057	0,690	0,751	0,073	0,710	0,758	**0,025**	0,710
		Hand + Arm	0,621	0,114	0,550	0,639	0,090	0,560	0,620	0,147	0,560
		Agent	0,484	0,260	0,460	0,383	0,606	0,470	0,408	0,579	0,470
		Agent – Hand	0,475	0,259	0,450	0,372	0,613	0,470	0,399	0,587	0,470
		Agent – Hand – Arm	0,485	0,245	0,450	0,392	0,548	0,460	0,415	0,529	0,460
	Silouhette	Hand	0,741	**0,029**	0,610	0,729	**0,039**	0,620	0,743	**0,018**	0,620
		Hand + Arm	0,609	0,091	0,510	0,608	0,100	0,530	0,596	0,148	0,530
		Agent	0,425	0,399	0,450	0,297	0,824	0,460	0,335	0,782	0,460
		Agent – Hand	0,399	0,468	0,440	0,278	0,851	0,460	0,312	0,824	0,460
		Agent – Hand – Arm	0,414	0,415	0,440	0,301	0,799	0,460	0,332	0,771	0,460
	Silouhette size	Hand	0,570	0,755	0,670	0,637	0,467	0,690	0,585	0,677	0,690
		Hand + Arm	0,588	0,362	0,610	0,579	0,429	0,620	0,568	0,393	0,620
		Agent	0,472	0,932	0,640	0,572	0,629	0,660	0,498	0,898	0,660
		Agent – Hand	0,471	0,940	0,650	0,593	0,553	0,660	0,508	0,890	0,660
		Agent – Hand – Arm	0,600	0,585	0,670	0,688	0,207	0,690	0,635	0,375	0,690
	Hu Moments	Hand	0,773	**0,001**	0,420	0,728	**0,005**	0,420	0,743	**0,003**	0,420
		Hand + Arm	0,816	**0,001**	0,570	0,865	**<0,001**	0,570	0,837	**<0,001**	0,570
		Agent	0,729	**0,009**	0,500	0,781	**0,002**	0,510	0,739	**0,005**	0,510
		Agent – Hand	0,793	**0,001**	0,420	0,815	**<0,001**	0,430	0,807	**<0,001**	0,430
		Agent – Hand – Arm	0,859	**<0,001**	0,570	0,837	**<0,001**	0,570	0,843	**<0,001**	0,570
temporal	Optic Flow	Hand	0,749	0,142	0,730	0,774	0,083	0,750	0,767	**0,047**	0,750
		Hand + Arm	0,701	**0,027**	0,560	0,683	**0,045**	0,580	0,684	**0,049**	0,580
		Agent	0,681	**0,025**	0,520	0,649	0,053	0,540	0,656	0,054	0,540
		Agent – Hand	0,331	0,923	0,520	0,443	0,635	0,540	0,377	0,873	0,540
		Agent – Hand – Arm	0,475	0,403	0,510	0,529	0,251	0,520	0,499	0,335	0,520
	HOF	Hand	0,844	**<0,001**	0,410	0,836	**<0,001**	0,410	0,851	**<0,001**	0,410
		Hand + Arm	0,771	**0,001**	0,430	0,800	**<0,001**	0,440	0,787	**0,001**	0,440
		Agent	0,827	**<0,001**	0,480	0,801	**0,001**	0,480	0,817	**<0,001**	0,480
		Agent – Hand	0,865	**<0,001**	0,450	0,776	**<0,001**	0,460	0,832	**<0,001**	0,460
		Agent – Hand – Arm	0,646	**0,031**	0,410	0,522	0,144	0,420	0,567	0,065	0,420
	Motion Energy	Hand	0,710	0,055	0,620	0,693	0,090	0,640	0,700	0,085	0,640
		Hand + Arm	0,620	0,057	0,490	0,564	0,153	0,510	0,577	0,153	0,510
		Agent	0,538	0,148	0,460	0,438	0,439	0,480	0,471	0,392	0,480
		Agent – Hand	0,460	0,387	0,480	0,501	0,283	0,490	0,462	0,480	0,490
		Agent – Hand – Arm	0,328	0,893	0,510	0,457	0,523	0,520	0,388	0,815	0,520
	Movement Direction	Hand	0,749	0,063	0,690	0,697	0,248	0,710	0,722	0,121	0,710
		Hand + Arm	0,616	0,238	0,600	0,663	0,119	0,610	0,625	0,231	0,610
		Agent	0,642	0,227	0,630	0,594	0,477	0,640	0,598	0,383	0,640
		Agent – Hand	0,677	0,137	0,640	0,590	0,533	0,650	0,624	0,280	0,650
		Agent – Hand – Arm	0,657	0,185	0,630	0,678	0,138	0,640	0,652	0,242	0,640
	Movement Velocity	Hand	0,637	0,068	0,530	0,619	0,096	0,540	0,608	0,139	0,540
		Hand + Arm	0,651	0,062	0,540	0,640	0,075	0,560	0,633	0,107	0,560
		Agent	0,544	0,512	0,590	0,620	0,226	0,610	0,569	0,507	0,610
		Agent – Hand	0,575	0,397	0,600	0,643	0,172	0,620	0,596	0,398	0,620
		Agent – Hand – Arm	0,545	0,248	0,520	0,567	0,205	0,540	0,526	0,386	0,540
high-level	I3D-K400		0,690	**0,020**	0,490	0,660	**0,050**	0,500	0,680	**0,040**	0,510
	TSN-K400		0,830	**<0,001**	0,620	0,870	**<0,001**	0,630	0,860	**<0,001**	0,640
	TSN-SSV2		0,810	**<0,001**	0,610	0,830	**<0,001**	0,630	0,830	**<0,001**	0,640
low-level	luminance		0,610	0,150	0,760	0,610	0,160	0,770	0,620	0,130	0,770
	contrast		0,500	0,730	0,650	0,610	0,270	0,660	0,550	0,550	0,660

Supplementary Table 5. Cosine similarity between monkey behavior and features from CNN layers with baselines derived from permutation tests.

			M1			M2			Group	
**CNNs**	**Layers**	**cos sim**	**p**	**base**	**cos sim**	**p**	**base**	**cos sim**	**p**	**base**
I3D-K400	1	0,783	**<0,001**	0,419	0,795	**<0,001**	0,433	0,800	**<0,001**	0,438
	2	0,871	**<0,001**	0,418	0,889	**<0,001**	0,433	0,892	**<0,001**	0,437
	3	0,898	**<0,001**	0,418	0,906	**<0,001**	0,434	0,914	**<0,001**	0,437
	4	0,896	**<0,001**	0,418	0,904	**<0,001**	0,433	0,912	**<0,001**	0,437
	5	0,781	**<0,001**	0,418	0,827	**<0,001**	0,433	0,815	**<0,001**	0,437
	6	0,773	**0,001**	0,418	0,809	**<0,001**	0,433	0,802	**<0,001**	0,438
TSN-K400	1	0,453	0,373	0,417	0,485	0,319	0,433	0,476	0,362	0,437
	2	0,479	0,294	0,417	0,483	0,326	0,433	0,488	0,322	0,437
	3	0,424	0,470	0,417	0,468	0,374	0,433	0,452	0,443	0,437
	4	0,463	0,344	0,417	0,579	0,095	0,433	0,528	0,206	0,437
	5	0,622	**0,038**	0,418	0,692	**0,009**	0,433	0,666	**0,018**	0,436
	6	0,902	**<0,001**	0,418	0,910	**<0,001**	0,433	0,918	**<0,001**	0,438
TSN-SSV2	1	0,493	0,254	0,418	0,514	0,236	0,434	0,510	0,252	0,437
	2	0,571	0,092	0,418	0,629	**0,039**	0,433	0,608	0,061	0,437
	3	0,856	**<0,001**	0,417	0,906	**<0,001**	0,433	0,893	**<0,001**	0,436
	4	0,894	**<0,001**	0,418	0,899	**<0,001**	0,433	0,909	**<0,001**	0,436
	5	0,842	**<0,001**	0,418	0,895	**<0,001**	0,433	0,880	**<0,001**	0,437
	6	0,850	**<0,001**	0,417	0,869	**<0,001**	0,432	0,871	**<0,001**	0,437
