# Supplementary material for: Dynamic spatiotemporal features in action recognition: a multimodal study
Source: Commun Biol. 2026 Apr 3;9:734. doi: 10.1038/s42003-026-09917-z (PMC13219507; doi:10.1038/s42003-026-09917-z)
Supplement: Supplementary file 2 — description of additional supplementary files [file 42003_2026_9917_MOESM2_ESM.pdf]

# Description of Additional Supplementary Files

**File name**: Supplementary Video 1

**Description**: Examples of the dynamic spatial-temporal features of the action videos. Each row indicates examples of grasping (upper), touching (middle), and reaching (bottom) actions, respectively. The first column displays examples of original action videos without the object and the background. The rest of columns indicate the dynamic spatial and temporal features (of the agent) correspond to the example action videos in the first column.
