# Supplementary material for: Dynamic spatiotemporal features in action recognition: a multimodal study
Source: Commun Biol. 2026 Apr 3;9:734. doi: 10.1038/s42003-026-09917-z (PMC13219507; doi:10.1038/s42003-026-09917-z)
Supplement: Supplementary file 4 — Reporting Summary [file 42003_2026_9917_MOESM4_ESM.pdf]

nature portfolio

Corresponding author(s): Ding Cui, Koen Nelissen

Last updated by author(s): Mar 12, 2026

# Reporting Summary

Nature Portfolio wishes to improve the reproducibility of the work that we publish. This form provides structure for consistency and transparency in reporting. For further information on Nature Portfolio policies, see our Editorial Policies and the Editorial Policy Checklist.

## Statistics

For all statistical analyses, confirm that the following items are present in the figure legend, table legend, main text, or Methods section.

| n/a | Confirmed | |
|---|---|---|
| ☐ | ☒ | The exact sample size (*n*) for each experimental group/condition, given as a discrete number and unit of measurement |
| ☐ | ☒ | A statement on whether measurements were taken from distinct samples or whether the same sample was measured repeatedly |
| ☐ | ☒ | The statistical test(s) used AND whether they are one- or two-sided<br>*Only common tests should be described solely by name; describe more complex techniques in the Methods section.* |
| ☐ | ☒ | A description of all covariates tested |
| ☐ | ☒ | A description of any assumptions or corrections, such as tests of normality and adjustment for multiple comparisons |
| ☐ | ☒ | A full description of the statistical parameters including central tendency (e.g. means) or other basic estimates (e.g. regression coefficient) AND variation (e.g. standard deviation) or associated estimates of uncertainty (e.g. confidence intervals) |
| ☐ | ☒ | For null hypothesis testing, the test statistic (e.g. *F*, *t*, *r*) with confidence intervals, effect sizes, degrees of freedom and *P* value noted<br>*Give P values as exact values whenever suitable.* |
| ☒ | ☐ | For Bayesian analysis, information on the choice of priors and Markov chain Monte Carlo settings |
| ☐ | ☒ | For hierarchical and complex designs, identification of the appropriate level for tests and full reporting of outcomes |
| ☐ | ☒ | Estimates of effect sizes (e.g. Cohen's *d*, Pearson's *r*), indicating how they were calculated |

*Our web collection on statistics for biologists contains articles on many of the points above.*

## Software and code

Policy information about availability of computer code

| | |
|---|---|
| Data collection | python |
| Data analysis | matlab, python, Blender, SPM12, JIP, TDT decoding toolbox, Inflated 3D ConvNets, Temporal Segment Networks, Kinetics 400 dataset, Something-Something V2 dataset, Detectron2 |

For manuscripts utilizing custom algorithms or software that are central to the research but not yet described in published literature, software must be made available to editors and reviewers. We strongly encourage code deposition in a community repository (e.g. GitHub). See the Nature Portfolio guidelines for submitting code & software for further information.

## Data

Policy information about availability of data

All manuscripts must include a data availability statement. This statement should provide the following information, where applicable:

- Accession codes, unique identifiers, or web links for publicly available datasets
- A description of any restrictions on data availability
- For clinical datasets or third party data, please ensure that the statement adheres to our policy

Data is partially available online on Zenodo: https://doi.org/10.5281/zenodo.18978813. Full data will be available from the corresponding author upon reasonable request.

## Research involving human participants, their data, or biological material

Policy information about studies with human participants or human data. See also policy information about sex, gender (identity/presentation), and sexual orientation and race, ethnicity and racism.

| | |
|---|---|
| Reporting on sex and gender | NA |
| Reporting on race, ethnicity, or other socially relevant groupings | NA |
| Population characteristics | NA |
| Recruitment | NA |
| Ethics oversight | NA |

Note that full information on the approval of the study protocol must also be provided in the manuscript.

# Field-specific reporting

Please select the one below that is the best fit for your research. If you are not sure, read the appropriate sections before making your selection.

☒ Life sciences ☐ Behavioural & social sciences ☐ Ecological, evolutionary & environmental sciences

For a reference copy of the document with all sections, see nature.com/documents/nr-reporting-summary-flat.pdf

# Life sciences study design

All studies must disclose on these points even when the disclosure is negative.

| | |
|---|---|
| Sample size | Our study used three macaques for fMRI and two of these three for behavioral training/testing. This sample size follows established practice in non-human primate systems neuroscience, where N=2–3 subjects with within-subject repeated measurements is standard due to ethical, logistical, and biological constraints. We designed the study as within-subject: for each animal we acquired >30 experimental sessions, yielding many repeated observations per condition. This approach provides high statistical power to detect stable effects at the individual level and to estimate reliability (session-wise variance and permutation tests) without relying on between-subject averaging.<br><br>A formal a priori power analysis is not applicable here because our primary outcomes are multivariate pattern discriminability (fMRI MVPA) and behavioral confusion structure, which depend on high-dimensional covariance and session-level stability rather than single-trial effect sizes. Instead, sample size targets were set from prior non-human primate fMRI/MVPA reports using 2–3 subjects with multiple sessions and from our previous studies showing that ≥30 sessions per subject yielded stable decoding estimates and narrow uncertainty on similarity metrics. Results are reported per subject with appropriate statistics across sessions, and key effects were replicated across animals, supporting sufficiency of the chosen sample size. |
| Data exclusions | For fMRI experiments, 39, 36, and 35 runs were collected respectively for three subjects, while 5, 1, and 1 run was excluded for each respective subject due to poor fixation performance (<90%). This exclusion criteria was decided beforehand to mainly keep the recording sessions with controlled eye movements, which might potentially influence the results of the study. |
| Replication | fMRI effects were reproduced within each monkey over >30 sessions/animal; all statistics are computed within subject across sessions and reported per animal. Behavioral results were obtained in two monkeys and reported per subject, providing subject-level replication.<br>For behavior and CNNs, we used held-out videos (generalization clips) never shown during training. Behavioral generalization included non-contingent reward on novel clips to prevent learning during testing. For fMRI MVPA, decoding used cross-validation (leave-out schemes) that prevent run/clip leakage.<br>Key behavior–model alignments were reproduced across three two-stream CNNs (I3D-K400, TSN-K400, TSN-SSV2).<br>For all cosine-similarity results we provide label-permutation nulls (baseline mean) and permutation p-values.<br>Analyses used predefined ROIs for AON and early visual cortex and a fixed preprocessing/analysis pipeline applied identically across animals and sessions; full parameters are detailed in the Methods/Supplement.<br>We document all analysis steps and parameter choices in the Methods and Supplement, so that key results can be independently checked.<br>Together, these measures—per-subject replication across many sessions, train–test segregation with held-out stimuli, architectural replication in models, and permutation-based baselines—are designed to verify that the findings are robust and reproducible rather than dependent on a particular dataset split, model, or analysis choice. |
| Randomization | Not applicable. This was a within-subject study; we did not assign animals to different experimental groups. Each monkey served as its own control across conditions. |
| Blinding | Given the within-subject design, investigator blinding was not required to prevent bias and is unlikely to affect the reported outcomes. |

# Reporting for specific materials, systems and methods

We require information from authors about some types of materials, experimental systems and methods used in many studies. Here, indicate whether each material, system or method listed is relevant to your study. If you are not sure if a list item applies to your research, read the appropriate section before selecting a response.

## Materials & experimental systems

| n/a | Involved in the study |
| --- | --- |
| ☒ | ☐ Antibodies |
| ☒ | ☐ Eukaryotic cell lines |
| ☒ | ☐ Palaeontology and archaeology |
| ☐ | ☒ Animals and other organisms |
| ☒ | ☐ Clinical data |
| ☒ | ☐ Dual use research of concern |
| ☒ | ☐ Plants |

## Methods

| n/a | Involved in the study |
| --- | --- |
| ☒ | ☐ ChIP-seq |
| ☒ | ☐ Flow cytometry |
| ☐ | ☒ MRI-based neuroimaging |

## Animals and other research organisms

Policy information about studies involving animals; ARRIVE guidelines recommended for reporting animal research, and Sex and Gender in Research

| | |
| --- | --- |
| Laboratory animals | Macaca mulatta, 5-7 years old |
| Wild animals | the study did not involve wild animals |
| Reporting on sex | the study included 3 male animals, sex information is irrelevant to the research aim of the study. |
| Field-collected samples | the study did not involve samples collected from the field |
| Ethics oversight | KU Leuven, Belgium |

Note that full information on the approval of the study protocol must also be provided in the manuscript.

## Plants

| | |
| --- | --- |
| Seed stocks | NA |
| Novel plant genotypes | NA |
| Authentication | NA |

## Magnetic resonance imaging

### Experimental design

| | |
| --- | --- |
| Design type | task-based, block design |
| Design specifications | 18 blocks per session, 30s per block |
| Behavioral performance measures | eye movement, mean percentage of fixation |

## Acquisition

| | |
|---|---|
| Imaging type(s) | functional |
| Field strength | 3 Tesla |
| Sequence & imaging parameters | gradient echo, EPI, fov=105x105x50mm, matrix size=84x84x40, slice thickness=1.25mm, TR=2 s, TE=17 ms, flip angle=90° |
| Area of acquisition | whole brain |

Diffusion MRI ☐ Used ☒ Not used

## Preprocessing

| | |
|---|---|
| Preprocessing software | SPM12, JIP |
| Normalization | nifti images, co-registration - both linear and non-linear |
| Normalization template | template anatomy (M12), Ekstrom et al., 2008 |
| Noise and artifact removal | When applied GLM, to account for head-motion and eye-movement related artifacts, six regressors corresponding to three rotations and translations along the x, y, and z-axis and three regressors corresponding to horizontal and vertical components of eye position and pupil diameter were included as covariates of no interest. |
| Volume censoring | NA |

## Statistical modeling & inference

| | |
|---|---|
| Model type and settings | GLM, fixed effect for individual subject<br>MVPA |
| Effect(s) tested | pair-wise decoding between conditions |

Specify type of analysis: ☐ Whole brain ☐ ROI-based ☒ Both

Anatomical location(s): we used independently defined ROIs from previous studies in the lab

| | |
|---|---|
| Statistic type for inference<br>(See Eklund et al. 2016) | NA |
| Correction | FWE |

## Models & analysis

| n/a | Involved in the study |
|---|---|
| ☒ | ☐ Functional and/or effective connectivity |
| ☒ | ☐ Graph analysis |
| ☐ | ☒ Multivariate modeling or predictive analysis |

Multivariate modeling and predictive analysis:
we applied pair-wise MVPA decodings for three action categories.
we used T-maps per run per condition as corresponding features for a given pair of decoding.
we applied leave-one-run-out approach for cross-validation in the pair-wise decoding.
Model performance was evaluated using decoding accuracy, averaged across all cross-validation folds.
